# Genetic Variants of *GPER/GPR30*, a Novel Estrogen-Related G Protein Receptor, Are Associated with Human Seminoma

**DOI:** 10.3390/ijms15011574

**Published:** 2014-01-21

**Authors:** Nicolas Chevalier, Rachel Paul-Bellon, Philippe Camparo, Jean-François Michiels, Daniel Chevallier, Patrick Fénichel

**Affiliations:** 1 Institut National de la Santé et de la Recherche Médicale (INSERM) UMR U1065/UNS, Centre Méditerranéen de Médecine Moléculaire (C3M), Equipe 5, Environnement, Reproduction et Cancers Hormono-Dépendants, Nice 06204, France; E-Mail: paul@unice.fr; 2 Institut Signalisation et Pathologie (IFR 50), Université de Nice-Sophia Antipolis, Faculté de Médecine, Nice 06000, France; 3 Centre Hospitalier Universitaire de Nice, Hôpital de l’Archet, Service d’Endocrinologie, Diabétologie et Médecine de la Reproduction, Nice 06000, France; 4 Hôpital Foch, Laboratoire d’Anatomie Pathologique, Suresnes 92151, France; E-Mail: anapath@hopital-foch.org; 5 Laboratoire d’Anatomie et Cytologie Pathologiques, Centre Hospitalier Universitaire de Nice, Hôpital Pasteur, Nice 06000, France; E-Mail: michiels.jf@chu-nice.fr; 6 Centre Hospitalier Universitaire de Nice, Hôpital de l’Archet, Service d’Urologie, Nice 06202, France; E-Mail: chevallier.d@chu-nice.fr

**Keywords:** GPER, GPR30, JKT-1 cells, estrogens, xeno-estrogens, testicular cancer, seminoma, polymorphisms, SNP, genetic susceptibility

## Abstract

Testicular germ cell tumors (TGCTs) are the most common solid cancers in young men, with an increasing incidence over several years. However, their pathogenesis remains a matter of debate. Some epidemiological data suggest the involvement of both environmental and genetic factors. We reported two distinct effects of estrogens and/or xeno-estrogens on *in vitro* human seminoma-derived cells proliferation: (1) an antiproliferative effect via a classical estrogen receptor beta-dependent pathway, and (2) a promotive effect via a non-classical membrane G-protein-coupled receptor, GPR30/GPER, which is only overexpressed in seminomas, the most common TGCT. In order to explain this overexpression, we investigated the possible association of polymorphisms in the *GPER* gene by using allele-specific tetra-primer polymerase chain reaction performed on tissue samples from 150 paraffin-embedded TGCT specimens (131 seminomas, 19 non seminomas). Compared to control population, loss of homozygous ancestral genotype GG in two polymorphisms located in the promoter region of GPER (rs3808350 and rs3808351) was more frequent in seminomas but not in non-seminomas (respectively, OR = 1.960 (1.172–3.277) and 7.000 (2.747–17.840); *p* < 0.01). These polymorphisms may explain GPER overexpression and represent a genetic factor of susceptibility supporting the contribution of environmental GPER ligands in testicular carcinogenesis.

## 1. Introduction

Testicular germ cell tumors (TGCT) are the most frequent cancer of young men with an increasing incidence all over the world [1], especially for seminoma tumors, the most frequent TGCT. The pathogenesis and reasons for this increase remain unknown but environmental factors, together with genetic susceptibility, have been suggested from epidemiological, clinical, and molecular data [2,3].

This environmental hypothesis, according to which fetal exposure to endocrine disruptors with estrogenic effects could participate to testicular germ cell carcinogenesis, by influencing the fate of germ stem cells (transformed gonocytes or undifferentiated spermatogonia) [4], supposes that TGCT are estrogen dependent. In fact, it is now well established that estrogens, the archetype of female hormones, contribute to the control of normal spermatogenesis [5]. 17β-estradiol (E_2_) is present at high concentrations in the adult testis due to testosterone conversion by aromatase [6], which is expressed by human germ cells [7]. Estrogens are able to stimulate proliferation of rat neonatal gonocytes *in vitro* [8] and to prevent apoptosis of human adult post-meiotic germ cells cultivated in preserved seminiferous tubules [9].

TGCT are considered to be raised from transformed gonocytes or undifferentiated spermatogonia [4]. Others, and we, have contributed to the concept of estrogen dependency of TGCT [10,11]. Seminoma tumors and seminoma cells both expressed functional aromatase, as well as estrogen receptor beta (ERβ), but not estrogen receptor alpha (ERα) [11,12]. Using the JKT-1 cell line derived from a human testicular seminoma [13], we have shown that E_2_ was able to inhibit human seminoma cell proliferation *in vitro* through an ERβ dependent mechanism [11], suggesting that ERβ acts on germ cells as a tumoral suppressor according to the observations performed on neonatal gonocytes of *ERβ*–KO mice [14]. However, when E_2_ was conjugated to bovine serum albumin (E_2_-BSA), which did not cross the membrane, the effect observed was completely different. Indeed, E_2_-BSA was able to promote JKT-1 cell proliferation *in vitro* by activating PKA and MAP kinases pathways due to a rapid phosphorylation of CREB transcription factor, involving a membrane G protein-coupled receptor (GPCR) [15]. We later identified this GPCR as GPR30 [16], a widely-conserved orphan GPCR, which has been recently renamed as G protein-coupled estrogen receptor (GPER) [17].

GPER is a seven-transmembrane domain protein, identified as a novel E_2_-binding protein structurally distinct from the classical estrogen receptors (ERα and ERβ). GPER can mediate rapid E_2_-induced non-genomic signaling events, including stimulation of adenylate cyclase and several other kinases [18]. Several hormone dependent cancers as breast, ovarian, and endometrium cancers express GPER. This expression also exhibits prognosis utility in such cancers [19–21] and GPER is able to modulate growth of hormonally responsive cancer cells *in vitro* [22,23]. Moreover, E_2_ has a low affinity for GPER, unlike some endocrine disruptors, such as bisphenol A or atrazine, which have a high affinity for GPER, as observed in ovarian and breast cancer cells [24,25], and recently in seminoma cells [26].

In testis, it is possible that this GPCR with no evident physiological ligand may interfere with estrogen and/or xeno-estrogen activation during normal and/or pathological regulation of germ cell proliferation and apoptosis [15,16]. It could also contribute to the malignant transformation of immature germ stem cells. Like other estrogen-dependent cancers, human seminoma express different estrogens receptors (here ERβ and GPER) and can be activated in different ways both by estrogens and xeno-estrogens depending on their respective affinity and cell microenvironment (receptor expression level, cofactors). In the current study, we investigated GPER expression in malignant human testicular germ cells (JKT-1 cell line), its ability to trigger *in vitro* seminoma cell proliferation, and the mechanisms involved in its overexpression in testicular carcinogenesis.

## 2. Results and Discussion

### 2.1. Localization of GPER in Human Seminoma-Derived Cells

GPER is a GPCR that induces rapid signaling through G_s_ or G_i_ proteins, strongly suggesting the plasma membrane as GPER’s site of action. However, the precise location of GPER remains controversial as alternately reported at the plasma membrane or in the endoplasmic reticulum.

**Figure 1.**
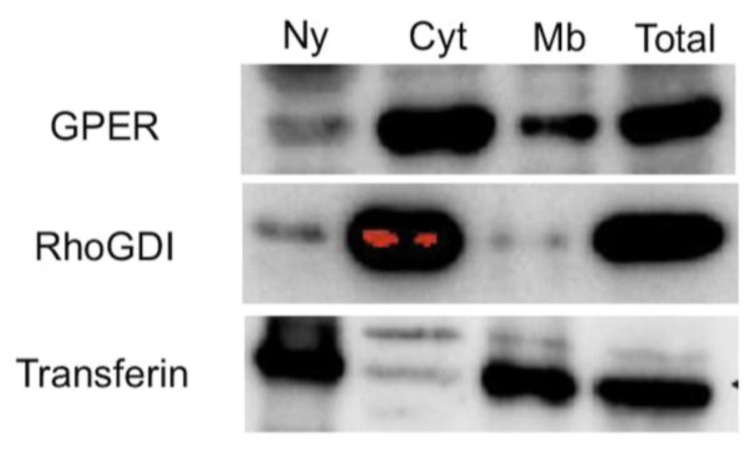
Subcellular fractionation of JKT-1 seminoma-derived cells. Transferin receptor (Transferin) was used as a tag of nuclear (Ny) and membrane (Mb) compartments, whereas RhoGDIα (Rho GDP-dissociation inhibitor) was used as a tag of cytoplasmic (Cyt) compartment.

As we previously reported [16], the co-localization of GPER with E_2_-BSA-FITC, which does not cross the membrane, strongly supported the membrane location of GPER in JKT-1 seminoma-derived cells. In order to assess the precise location of GPER in seminoma-derived cells, we performed a subcellular fractionation using a sucrose gradient centrifugation (Figure 1). Our experiments showed that GPER was, indeed, located at the cell membrane but also in the cytoplasm, with a ratio of approximately 20%–80%. This finding is in agreement with other reports [27–30] and suggests that an intense cell trafficking of the protein depending on the cell microenvironment probably exists, as observed with other GPCR [31], rather than suggesting a problem due to the different antibodies used, which triggered different epitopes (intra- or extracytoplasmic) [27–30].

### 2.2. Role of GPER in Human Seminoma-Derived Cells Proliferation

**Figure 2.**
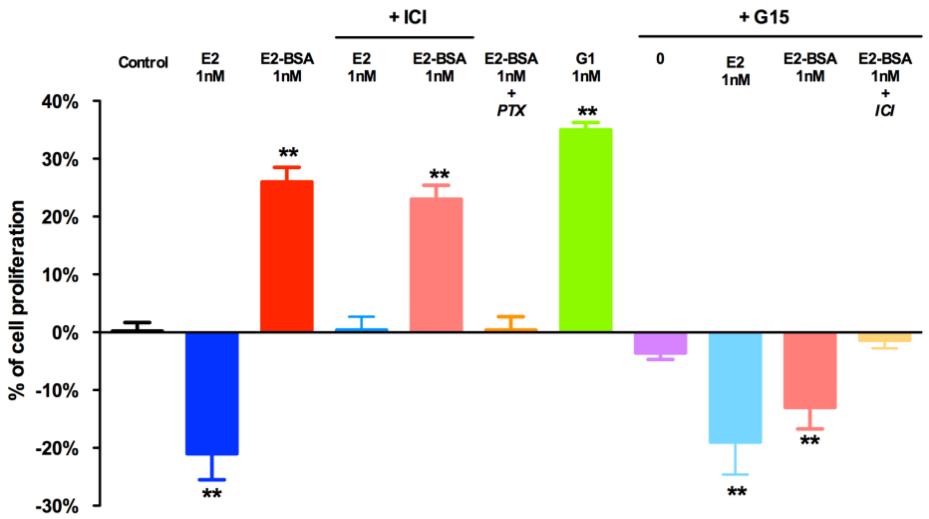
Analysis of JKT-1 cell proliferation *in vitro*. JKT-1 cells were seeded in six-well plates (0.6 × 10^6^ cells/well). After 48 h, the JKT-1 cells were washed and estrogen starved overnight in phenol red-free DMEM (Dulbeccos’s Modified Eagle Medium) supplemented with 1% charcoal-stripped fetal bovine serum. Serum-deprived JKT-1 cells were then incubated for 24 h with E_2_-BSA (1 nM), after a pre-treatment with G15 (1 nM) and/or ICI-182,780 (1 μM) or Pertussis toxin (100 ng/mL; PTX). G1 (1 nM) was used as a positive control. Histograms represent percentages of variation in the JKT-1 cell number compared with the control (0%); all results are expressed as means ± standard error of the mean (SEM) of at least five independent experiments. ** *p* < 0.01.

After 24-h exposure at a physiological intra-testicular concentration of 10^−9^ M, E_2_ induced a significant decrease in cell proliferation, whereas E_2_-BSA, at the same concentration, stimulated JKT-1 cell proliferation [15]. As we previously reported, this E_2_-BSA specific effect was not inhibited by ICI-182,780, a pure ER antagonist, but was reversed by *Pertussis toxin*, a G protein inhibitor [15], we hypothesized that E_2_-BSA directly interacted with GPER to induce JKT-1 cell proliferation (Figure 2).

G1, a GPER-selective agonist, reproduced the same proliferative effect as that observed with E_2_-BSA. G15, a GPER-selective antagonist, had no effect alone on JKT-1 cell proliferation but when it was added to E_2_-BSA, we observed the same anti-proliferative effect than obtained with E_2_ alone. Co-addition of G15 and ICI-182,780 completely neutralized the E_2_-BSA-induced proliferative effect, suggesting that GPER mediated the membrane proliferative effects of estrogens, whereas the effect observed with G15 and E_2_-BSA was in fact due to a part of free E_2_ in the E_2_-BSA mixture (Figure 2).

### 2.3. Overexpression of GPER in Human Seminomas

#### 2.3.1. Relative Expression of GPER in Distinct TGCT

**Figure 3.**
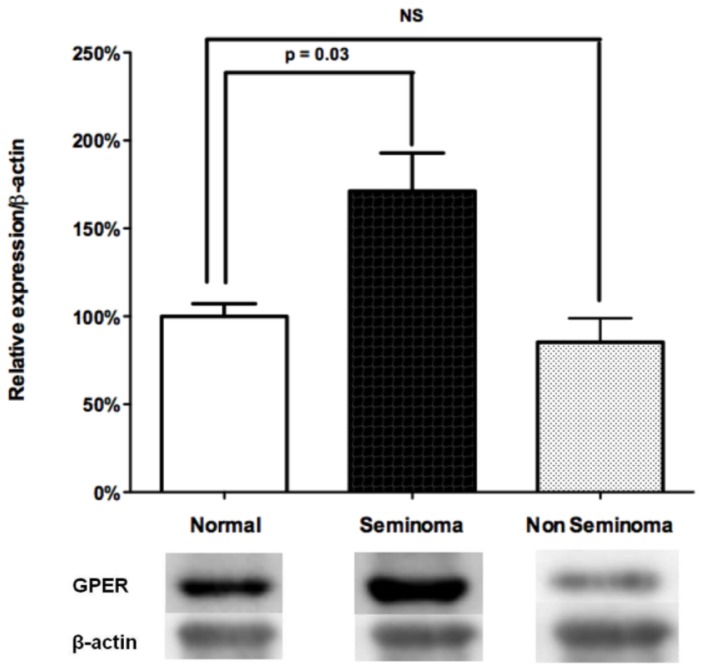
Analysis of relative expression of GPER protein (Western blotting) in seminomas (*n* = 8) and in non-seminoma tumors (*n* = 7) compared with the peri-tumoral normal testicular tissue for each patient (represented as 100%). Results are expressed as means ± S.D.; *β-actin* was taken in each case as a house-keeping gene; NS: non significant. Adapted from Chevalier *et al*. [16].

As reported by Franco *et al*. [32], and Rago *et al*. [33], we recently demonstrated that GPER was overexpressed in seminomas but not in non-seminoma tumors by using a robust quantitative approach of mRNAs and proteins levels (Figure 3), and comparison with the peri-tumoral normal testicular tissue for each patient (excluding inter-individual variations of GPER expression as each patient represents its own control) [16].

**Figure 4.**
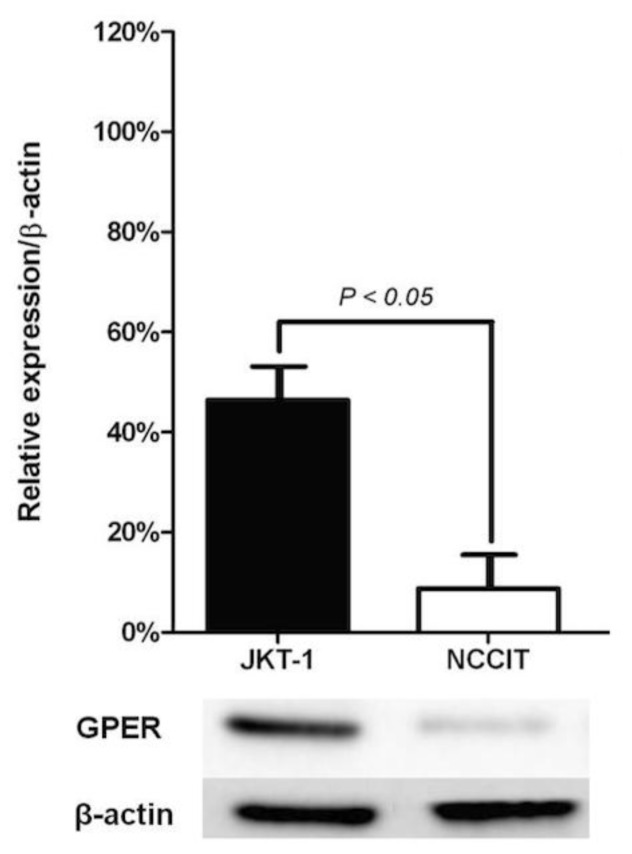
Relative expression of GPER protein (Western blotting) in human malignant testicular germ cell lines (JKT-1, a human testicular seminoma-derived cell line; NCCIT, a human testicular embryonic carcinoma cell line). Results are expressed as means ± standard error of the mean (SEM) of three different experiments; *β-actin* was taken in each case as a house-keeping gene.

In order to confirm the selective overexpression of GPER in seminomas, we compared expression pattern of JKT-1 (seminoma-derived) and NCCIT (choriocarcinoma) cells by Western blotting and RT-PCR (Figure 4). Both, JKT-1 and NCCIT cells expressed GPER, but JKT-1 cells showed significantly higher GPER protein levels than the NCCIT cells (*p* < 0.05). Further investigations on other germ cell tumor derived cellular lines are needed to confirm our observation.

#### 2.3.2. Mechanisms Leading to Overexpression of GPER in Human Seminomas

Such a GPER overexpression has been already linked to the development of advanced breast cancer [21], high-grade endometrial tumors [19], and poor prognosis for ovarian cancer [20]. Thus, it is critical to determine the precise mechanisms leading to such a GPER overexpression in seminomas and to assess its role in testicular carcinogenesis considering the ability for xeno-estrogens to promote seminoma cell proliferation [26,34].

**Figure 5.**
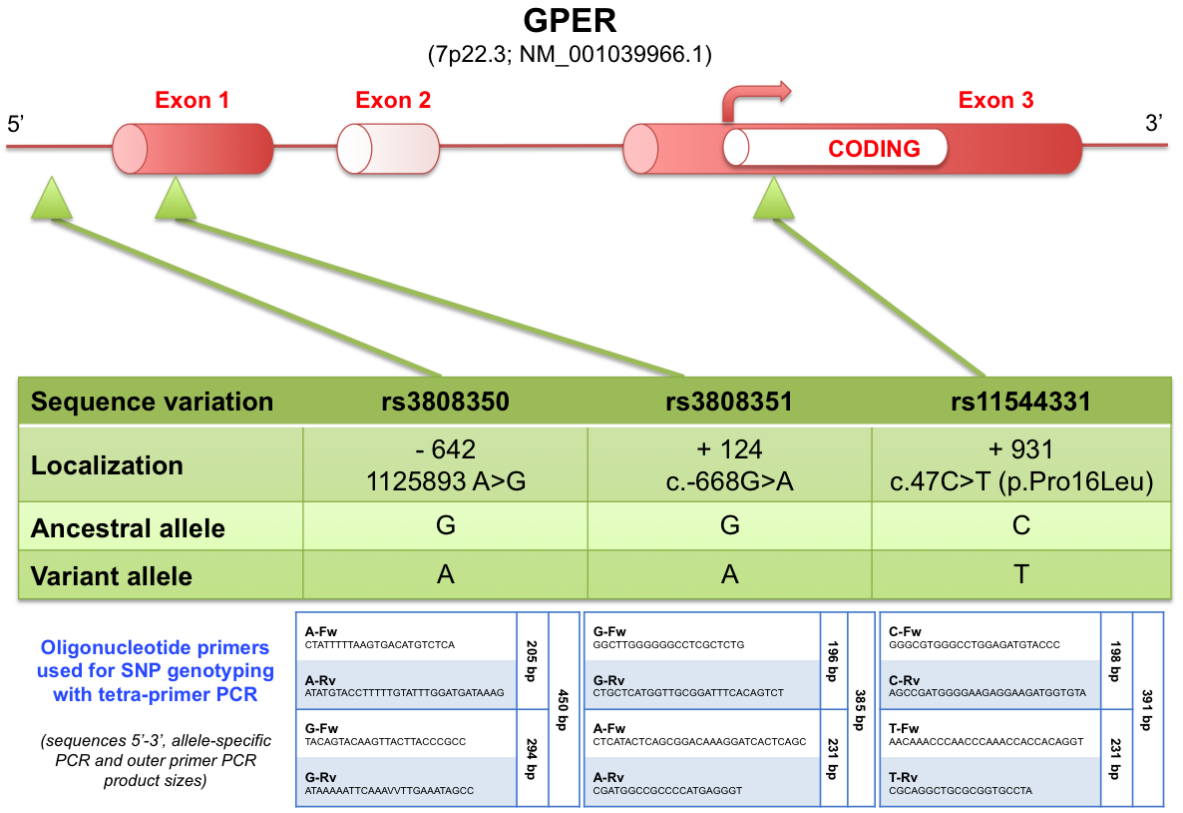
Localization of the three genotyped *GPER* SNPs with their relative position from the transcription start site and the oligonucleotide primers used for SNPs genotyping with tetra-primer PCR. Ancestral and variant alleles were determined in the HapMap project and reported on the internet database [37]. Filled boxes: untranslated region; open box: coding region; curved arrow: translation start site.

One explanation for this overexpression could be single nucleotide polymorphisms (SNPs), which are the most frequent genetic variations in genomic sequences and are widely associated with human diseases [35] and TGCTs [36]. Among the 40 SNPs identified in the *GPER* gene (GenBank accession no. NM_001039966), only four were reported to be associated with human neoplasms, according to the internet database [37] The first of these four SNPs has been reported in gastric cancer [38] and creates an alternative splice site that produces a frameshift protein with no cellular relevance. The other three SNPs are associated with aggressive histopathological characteristics of breast cancers [39]. These three SNPs have higher biological relevance, with two of them being located in the 5′ region of the *GPER* gene (SNP rs3808350 located in the 5′-regulatory region and SNP rs3808351 located in the 5′-untranslated region and containing the gene promoter), which might affect its expression levels (Figure 5). The third SNP results in an amino acid exchange (p.Pro16Leu) in the only coding exon (exon 3) of the *GPER* gene, which might alter GPER protein structure and function.

Thus, we investigated the *GPER* genotype, its polymorphisms and its correlation with clinical and histopathological characteristics in a large cohort of 150 Caucasian patients who underwent surgeries for testicular cancer between 1995 and 2011 at four surgical centers (classic seminoma: *n* = 131; non seminoma: *n* = 19).

The mean concentration of DNA isolated per extraction was 72.7 μg (range, 12–258 μg). As previously reported [40], we observed an extensive degradation of the DNA due to its source, especially for non seminomas. Due to this degradation, genotyping could only be performed in 89 patients for SNP rs3808350 (81 seminomas and eight non-seminomas, respectively), 100 patients for SNP rs3808351 (91 and nine) and 123 patients for SNP rs11544331 (115 and eight), leading to genotyping success rates of 61.8%, 69.5%, and 87.8% for seminomas and 42.1%, 47.3%, and 42.3% for non-seminomas, respectively. Nevertheless, we performed a control sequencing, for a subgroup of patients, between DNA obtained from paraffin-embedded primary tumors and DNA obtained from fresh whole blood and found no difference, especially no loss of heterozygoty (LOH) in tumoral DNA (data not shown).

We first analyzed a missense SNP (rs11544331; (c.47 C > T), located in the only coding exon (exon 3) of *GPER* gene, which leads to an amino-acid (p.Pro16Leu) exchange. Such an exchange might alter GPER protein structure and its function, but not its expression. Thus, this SNP may not contribute to GPER overexpression, previously observed in seminomas. As expected, allele and genotype frequencies were not different between neoplastic patients and control population for both seminomas and non-seminomas (Table 1). We also investigated genotypes of JKT-1 seminoma-derived cells and NCCIT choriocarcinoma-derived cells, which were not different from the control population (CC and CC).

In contrast, the other two *GPER* SNPs tested were strongly associated with seminomas, but not with non seminomas (Table 1). The homozygous AA genotype of the SNP rs3808350 in the 5′-untranslated region and the SNP rs3808351 in the 5′-regulatory region were both significantly more common in seminoma patients than in the control population, suggesting that homozygous ancestral genotype GG could exert relative protective effects on tumor development. These data are consistent with the location of these two SNPs, which allows them to directly modulate GPER protein expression. As they are located in the promoter region of *GPER* gene, these two polymorphisms could explain the GPER overexpression previously reported in such testicular tumors [16,32,33]. Nevertheless, this hypothesis should be confirmed by comparing, for each patient, the relation between SNP genotype and the precise level of GPER protein expression in a larger cohort. It would be also necessary to verify whether these two SNP affect or not the function of this receptor. We investigated the genotypes of JKT-1 seminoma-derived cells and NCCIT choriocarcinoma-derived cells for these two SNPs. We found no difference from the control population for SNP rs3808350 (genotype AG), but an homozygous carriage of minor allele A for SNP rs3808351, as more frequently observed in the tumoral samples; this genotype could also explain *GPER* expression in germ cell tumors. However, JKT-1 and NCCIT cells exhibited the same genotype for the two SNPs, suggesting that protein overexpression in seminoma could be linked to a post-translational regulation, involving several factors as small RNAs.

We obtained clinical data from 56 genotyped patients with seminomas. The allele and genotype frequencies of each SNP in these patients were not significantly different from those observed in the entire neoplastic population, and from those observed in the reference population (Table 2). The mean age at diagnosis was 38.6 ± 9.5 years. The mean tumor size was 46.2 ± 20.4 mm, with tumor spread beyond testis according to TNM (Tumor, Nodes, Metastasis) classification (≥pT2) in 20 of 56 cases (35.7%). We did not find a correlation between a specific genotype and tumor characteristics (age at diagnosis, tumor size, or tumor spread) for these patients.

## 3. Experimental Section

### 3.1. Cell Culture

The JKT-1 cell line, a kind gift from Dr. Keigo Kinugawa, was established from a human pure testicular seminoma developed from the testis of a 40-year-old man [13]. It was recently verified that the JKT-1 cells maintained in our laboratory still expressed specific embryonic stem cell markers [41]. The JKT-1 cells were maintained in DMEM (Invitrogen^®^, Carlsbad, CA, USA) supplemented with 2% sodium pyruvate and 10% FBS (Invitrogen^®^, Carlsbad, CA, USA) in a humidified 5% CO_2_ atmosphere at 37 °C. The NCCIT cell line was developed from a human testicular embryonic carcinoma and obtained from the American Type Culture Collection (Manassas, VA, USA). These TGCT adherent cells were grown in RPMI-1640 medium (Invitrogen^®^, Carlsbad, CA, USA) supplemented with 15% FBS and were maintained in a humidified 5% CO_2_ atmosphere at 37 °C.

### 3.2. Subcellular Fractionation

JKT-1 cells were grown in 10-cm dishes at a density of 4.9 × 10^6^ cells per dish. After 48 h, the JKT-1 cells were scraped in 5 mL cold PBS, pelleted and homogenized in 250 μL cold SI buffer (250 mM sucrose, 3 mM imidazole, pH 7.4, 1 mM PMSF protease inhibitor). Cells were lysed by passing 40 times through a 25G needle (U-100 Insulin, Terumo^®^, Somerset, NJ, USA). Nuclei were removed by centrifugation for 10 min at 10,000 *g* at 4 °C. Protein concentration of the post-nuclear supernatants (PNS) was normalized. PNS were centrifuged for 1 h at 100,000 *g* at 4 °C. Supernatants correspond to the cytosolic fraction. Pellets, homogenized in an equal volume of SI buffer, correspond to membranes.

Equal amounts (30 μL) of each fraction were resolved on a 12% SDS-polyacrylamide gel. The proteins were transferred to a polyvinylidene difluoride membrane (Immobilon P; Millipore™, Billerica, MA, USA), probed with anti-GPER Ab (Santa Cruz Biotechnology^®^, Santa Cruz, CA, USA) and with anti-Rho-GDIα (Santa Cruz Biotechnology^®^, Santa Cruz, CA, USA) and anti-transferrin receptor (Invitrogen^®^) Abs to control fractionation, then detected using HRP-linked secondary Ab and the ECL System (GE Healthcare^®^, Chalfont St. Giles, UK). All experiments were performed in triplicate and the blots shown are representative.

### 3.3. Cell Proliferation Assay

JKT-1 cells were seeded in six-well plates (0.6 × 10^6^ cells/well). After 48 h, the JKT-1 cells were washed and oestrogen starved overnight in phenol red-free DMEM supplemented with 1% charcoal-stripped FBS. We then added E2 (Sigma-Aldrich^®^, Saint Louis, MO, USA), freshly prepared E2-BSA (Sigma-Aldrich^®^, Saint Louis, MO, USA) devoid of free E2, which is removed by filtration, ICI-182,780 (fulvestrant; Falsodex^®^, AstraZeneca, Wilmington, DE, USA), G1 (Calbiochem^®^, Merck KGaA, Darmstadt, Germany), G15 (kindly supplied by Eric R. Prossnitz) [42], or ethanol (as a vehicle control) at 10^−9^ M concentration [15,43], and incubated them for 24 h. We harvested the cells using trypsin and counted them using the Vi-CELL software (Beckman Coulter, Fullerton, CA, USA). Results are expressed as percentages of variation compared with the control.

### 3.4. Protein Purification and Western Blotting of Cells and Tumor Samples

The cells were grown in 10-cm dishes at a density of 4.9 × 10^6^ cells per dish. After 48 h, the cells were washed with PBS, and the cell pellets were lysed in ice-cold lysis buffer Brij96/Nonidet P-40 (50 mM Tris HCl (pH 7.5), 1% Nonidet P-40, 1% Brij96 (Fluka^®^ AG, Buchs, Switzerland), 1 mM Na_3_VO_4_, 10 mM β-glycerophosphate, 10 mM NaF, 2 mM EDTA and protease inhibitors (Complete™; Roche Diagnostics, Indianapolis, IN, USA). The lysates were sonicated twice for 7 s on ice and centrifuged for 15 min at 14,000 rpm.

Control and malignant human tests were collected from patients who underwent orchidectomy for TGCT (seminoma (*n* = 8) and non-seminoma (*n* = 7)) and who gave informed consent. The samples were frozen at −80 °C before being ground in cold Tris (10 mM, pH 7.4) containing protease inhibitors.

Protein concentrations of the cell and tissue lysates were determined by the Bradford method. Equal amounts of the whole protein extract were resolved on a 12% SDS-polyacrylamide gel. The proteins were transferred to a polyvinylidene difluoride membrane (Immobilon P; Millipore™, Billerica, MA, USA), probed with anti-GPER Ab (Santa Cruz Biotechnology^®^, Santa Cruz, CA, USA), and detected using HRP-linked secondary Ab and the ECL System (GE Healthcare^®^, Chalfont St. Giles, UK). After the blots were stripped, we verified equal loading of the protein by reprobing the same blots with anti-actin Ab (Cell Signaling Technology^®^, Danvers, MA, USA). All experiments were performed in triplicate and the blots shown are representative.

All data were analyzed using the StatView^®^5 software (SAS Institute Inc., Cary, NC, USA). Results of the cell count and densitometric analysis are expressed as percentages of variation compared with the control. A non-parametric Mann–Whitney *U* test was used for statistical analysis. All probabilities were two-sided and *p* < 0.05 was considered statistically significant.

### 3.5. SNP Analysis of Tumor Samples

Tumor specimens were obtained from 150 Caucasian patients who underwent surgeries for testicular cancer between 1995 and 2011 at four surgical centers (Centre Hospitalier Universitaire de Nice; Hospices Civils de Lyon; Hôpital Foch, Suresnes and Hôpital d’Instruction des Armées du Val de Grâce, Paris, France). All tumor specimens were embedded in paraffin. According to the World Health Organization classification, histological diagnosis of classic seminoma of young men, excluding spermatocytic seminoma in older men, was established in 131 patients; the 19 others patients were diagnosed as non seminomas, including choriocarcinoma embryonal cell carcinoma and teratoma. All subjects gave written consent to participate in the study, which was approved by the local Ethics Committee. Genomic DNA extraction was performed using 10-μm-thick serial sections cut from each specimen block, as previously reported [40].

The DNA-containing lysate was subjected to tetra-primer ARMS-PCR to achieve allele-specific amplification [35] by using primers listed in Figure 5 (synthesised by Eurogentec™, Liège, Belgium) and QIAGEN^®^ Multiplex PCR Plus Kit (Qiagen Inc., Valencia, CA, USA). Polymerase Chain Reaction (PCR) mixture contained 0.2 μM final concentration of each primer and 100 ng genomic DNA and was prepared according to the manufacturer’s instructions. PCR was performed using Thermocycler Q-Cycler II (Quanta Research™, Taoyuan County, Taiwan) by using the following reaction conditions: initial activation at 95 °C for 5 min; 35 cycles of denaturation at 95 °C for 30 s, annealing at 60 °C for 90 s and extension at 72 °C for 30 s; and final extension at 68 °C for 10 min. PCR products were analyzed by electrophoresis on 2% agarose gel in 1× TAE buffer (50 mM Tris-HCl (pH 8.0), 20 mM sodium acetate and 2 mM EDTA) and stained with GelRed™ Nucleic Acid Stain (Biotium Inc., Hayward, CA, USA). All experiments were performed in triplicate, and the data shown are representative of 3 experiments. Allele-specific PCR product sizes were as follows: 205/294 bp (A/G) for SNP rs3808350, 231/196 bp (A/G) for SNP rs3808351, and 198/231 bp (C/T) for SNP rs11544331.

SNP frequencies were compared with genotype frequencies determined in the HapMap project and reported on the internet database [37] as follows: for SNP rs3808350, genotype frequencies are 0.366 (AA), 0.491 (AG), and 0.143 (GG), and allele frequencies are 0.612 (A) and 0.388 (G); for SNP rs3808351, genotype frequencies are 0.054 (AA), 0.514 (AG), and 0.432 (GG), and allele frequencies are 0.311 (A) and 0.689 (G); and for SNP rs11544331, genotype frequencies are 0.650 (CC), 0.302 (CT), and 0.048 (TT), and allele frequencies are 0.801 (C) and 0.199 (T).

Deviation from the Hardy–Weinberg equilibrium between normal and neoplastic populations was estimated using chi-square test. Statistical tests for association (with 95% confidence interval) and significance were performed using StatView^®^5 software (SAS Institute Inc., Cary, NC, USA). Odds ratio (OR) was calculated using the more frequent homozygous genotypes as reference group. A *p* value < 0.05 was considered statistically significant.

## 4. Conclusions

Several research groups have recently shown that GPER (GPR30), an orphan GPCR with no evident physiological ligand, mediates a rapid E_2_-dependent activation of signal transduction pathways in various human estrogen-dependent cancer cells (breast, ovary and endometrium) and displays E_2_ binding typical of a membrane oestrogen receptor [22,28,44,45]. We confirmed, in the present study, that GPER was overexpressed in seminomas, was localized at the membrane of human seminoma cells and was able to mediate the promotive effect on seminoma cell proliferation observed *in vitro* with E_2_-BSA.

Despite a quite limited neoplastic cohort, we reported for the first time a significant genotype-phenotype association between *GPER* SNP and seminomas. One can suppose that the presence of such polymorphisms represent a genetic factor of susceptibility able to increase the risk of endocrine disruption, for example foetal exposure to bisphenol A, which seems to carry a high affinity for GPR30 expressed by germ stem cells [34]. Indeed, the preservation of these ancestral alleles may also represent a prognosis factor for seminomas. As our cohort was too small, we were not able to identify a clear correlation between these two SNP and clinical and histopathological characteristics; then we could not definitively consider these genotypes as protective markers. Nevertheless, it could become a potential marker for screening testicular germ cell cancer in patients with high risk factors (as cryptorchidism or infertility). These different points are now under investigation in our laboratory.

## Figures and Tables

**Table 1.** Analysis of *GPER* single nucleotide polymorphisms (SNP) frequencies in seminoma and non-seminomas tissues. Allele and genotype frequencies were determined after performing tests for deviation from the Hardy-Weinberg equilibrium. “*n*” is the total number of samples in each category.

		Genotype frequency			Allele frequency	
	
Sequence variation (localisation)		AA	AG	GG	A	G
rs3808350 (1125893 A > G)	Reference population (*n* = 224)	0.366 (*n* = 82)	0.491 (*n* = 110)	0.143 (*n* = 32)	0.612 (*n* = 274)	0.388 (*n* = 174)
	Seminoma (somatic) (*n* = 81)	0.531 (*n* = 43)	0.444 (*n* = 36)	0.025 (*n* = 2)	0.753 (*n* = 122)	0.247 (*n* = 40)
	*p*	0.0122	0.5174	0.0032		0.0011
	Non seminoma (somatic) (*n* = 8)	0.250 (*n* = 2)	0.625 (*n* = 5)	0.125 (*n* = 1)	0.562 (*n* = 9)	0.438 (*n* = 7)
	*p*	0.7142	0.4973	1.0000		0.7956

rs3808351 (c.-668 G > A)	Reference population (*n* = 222)	0.054 (*n* = 12)	0.514 (*n* = 114)	0.432 (*n* = 96)	0.311 (*n* = 138)	0.689 (*n* = 306)
	Seminoma (somatic) (*n* = 91)	0.154 (*n* = 14)	0.670 (*n* = 61)	0.176 (*n* = 16)	0.489 (*n* = 89)	0.511 (*n* = 93)
	*p*	0.0061	0.0123	<0.0001		<0.0001
	Non seminoma (somatic) (*n* = 9)	0.111 (*n* = 1)	0.889 (*n* = 8)	0.000 (*n* = 0)	0.555 (*n* = 10)	0.445 (*n* = 8)
	*p*	0.4119	0.0381	0.0114		0.0385

**Sequence variation (localisation)**		**CC**	**CT**	**TT**	**C**	**T**

rs11544331 (c.47 C > T; p.Pro16Leu)	Reference population (*n* = 4374)	0.65 (*n* = 2843)	0.302 (*n* = 1321)	0.048 (*n* = 210)	0.801 (*n* = 7007)	0.199 (*n* = 1741)
	Seminoma (somatic) (*n* = 115)	0.617 (*n* = 71)	0.365 (*n* = 42)	0.018 (*n* = 2)	0.800 (*n* = 184)	0.200 (*n* = 46)
	*p*	0.5328	0.1763	0.1918		0.9627
	Non seminoma (somatic) (*n* = 8)	0.875 (*n* = 7)	0.125 (*n* = 1)	0.000 (*n* = 0)	0.937 (*n* = 15)	0.063 (*n* = 1)
	*p*	0.3358	0.4812	0.8468		0.2921

**Table 2.** Analysis of clinical data of the 56 genotyped patients with seminomas according to the *GPER* genotype. Data are expressed as mean ± standard deviation or as percentage; “*n*” is the total number of genotyped samples in each category. Comparisons were performed between each genotype for each SNP; a *p* value < 0.05 was considered statistically significant (NS: non significant).

		Age at diagnosis		Tumoral size		Tumoral Spread	
		
Sequence variation (localisation)		Age (years)	*p*	Size (mm)	*p*	≥pT2	*p*
**rs3808350** (1125893 A > G)	**AA** (*n* = 26)	39.62 ± 9.26	NS	46.08 ± 20.20	NS	26.9% (7/26)	NS
	**AG** (*n* = 19)	39.68 ± 10.50	NS	41.00 ± 19.50	NS	31.6% (6/19)	NS
	**GG** (*n* = 1)	38.00 ± 0.00	NS	60.00 ± 0.00	NS	100% (1/1)	NS

**rs3808351** (c.-668 G > A)	**AA** (*n* = 12)	37.58 ± 7.87	NS	50.00 ± 21.10	NS	50.0% (6/12)	NS
	**AG** (*n* = 32)	39.16 ± 9.83	NS	43.56 ± 19.91	NS	28.1% (9/32)	NS
	**GG** (*n* = 8)	40.13 ± 10.12	NS	43.75 ± 25.03	NS	25.0% (2/8)	NS

**rs11544331** (c.47 C > T; p.Pro16Leu)	**CC** (*n* = 36)	37.31 ± 8.78	NS	49.83 ± 20.35	NS	36.1% (13/36)	NS
	**CT** (*n* = 20)	40.70 ± 11.01	NS	41.40 ± 19.35	NS	20.0% (4/20)	NS
	**TT** (*n* = 0)	-	-	-	-	-	-
